# Survival disparities in HER2-low versus HER2-positive breast cancer: a systematic review of progression-free survival and overall survival

**DOI:** 10.3389/fonc.2026.1839945

**Published:** 2026-09-10

**Authors:** Widyanti Soewoto, Ida Bagus Budhi S. A., Wibisono, Kristanto Yuli Yarso, Joko Wibowo Sentoso

**Affiliations:** 1 Department of Surgical Oncology, Faculty of Medicine, Sebelas Maret University, Dr. Moewardi Hospital, Surakarta, Indonesia; 2 Department of Digestive Surgery, Faculty of Medicine, Sebelas Maret University, Dr. Moewardi Hospital, Surakarta, Indonesia; 3 Department of Urology, Faculty of Medicine, Sebelas Maret University, Dr. Moewardi Hospital, Surakarta, Indonesia; 4 Department of Surgery, Faculty of Medicine, Sebelas Maret University, Dr. Moewardi Hospital, Surakarta, Indonesia

**Keywords:** breast cancer, HER2-low, HER2-positive, overall survival (OS), progression-free survival (PFS)

## Abstract

**Background:**

The emergence of HER2-low breast cancer as a distinct clinical entity has challenged the traditional binary classification of HER2 status. This systematic review aims to evaluate the disparities in Progression-Free Survival (PFS) and Overall Survival (OS) between HER2-low and HER2-positive breast cancer patients.

**Methods:**

A systematic search was performed across PubMed, Scopus, Cochrane Library, ESMO and ASCO for studies published through January 2026. The Clinical trials and observational studies reporting Progression-Free Survival (PFS) and Overall Survival (OS) with Hazard Ratios (HR) were included. Due to significant clinical and methodological heterogeneity, a formal meta-analysis was deemed inappropriate; consequently, evidence was synthesized qualitatively following the Synthesis Without Meta-analysis (SWiM) guidelines. The protocol was prospectively registered in PROSPERO (CRD420261293004).

**Results:**

A total of 536 records were identified; after removing 356 duplicates, 180 were screened, and 10 trials met the inclusion criteria. Preliminary synthesis indicates that HER2-low tumors exhibit distinct survival trajectories compared to HER2-positive cohorts, often influenced by Hormone Receptor (HR) co-expression and the type of targeted therapy received.

**Conclusion:**

Understanding survival disparities is crucial for optimizing treatment sequencing in the era of antibody-drug conjugates (ADCs).

**Systematic review registration:**

https://www.crd.york.ac.uk/prospero/, identifier CRD420261293004.

## 1 Introduction

The clinical management of breast cancer has long been anchored in the binary classification of Human Epidermal Growth Factor Receptor 2 (HER2) status, which historically categorized tumors as either HER2-positive or HER2-negative to determine eligibility for trastuzumab-based therapies (1). The traditional binary framework of HER2 assessment is being superseded by the recognition of HER2-low status clinically categorized by IHC 1+ or 2+/ISH-negative scores marking a significant departure from historical diagnostic standards (2). Representing approximately 45–55% of all breast cancer cases, this entity has challenged the traditional diagnostic dichotomy and established itself as a distinct clinical subset with unique therapeutic implications (2, 4).

The clinical relevance of HER2-low expression has been fundamentally reshaped by the success of second-generation antibody-drug conjugates (ADCs), most notably Trastuzumab Deruxtecan (T-DXd). The efficacy of these novel ADCs in HER2-low and even HER2-ultralow tumors as evidenced by the landmark DESTINY-Breast04 and DESTINY-Breast06 trials is attributed to the bystander effect, which enables potent antitumor activity despite low or heterogeneous target density. Furthermore, ongoing evaluations in the DESTINY-Breast07 trial are expanding the use of these agents into earlier lines of therapy, highlighting a shift where HER2-low status serves as a critical predictive biomarker for targeted payloads rather than merely a prognostic indicator (3, 30, 31). Despite these advancements, the prognostic significance of HER2-low expression relative to the established HER2-positive phenotype remains a subject of intense debate (10). While HER2-positive disease exhibits distinct oncogenic dependency, it remains highly responsive to established, multi-line therapeutic protocols utilizing dual-HER2 blockade (8, 11), HER2-low tumors often exhibit biological behavior more closely aligned with HER2-zero subtypes, where survival is heavily influenced by Hormone Receptor (HR) co-expression (7, 13). Intratumoral heterogeneity in low-level HER2 expression presents a major challenge for diagnostic accuracy, potentially leading to discrepancies in single-biopsy results (21, 26).

Despite the increasing volume of literature on HER2-low breast cancer, existing studies often present conflicting results regarding its independent prognostic value. Most current reviews focus predominantly on the efficacy of novel ADCs, yet there remains a limited of systematic evidence that directly compares the survival outcomes of HER2-low patients against the HER2-positive population, particularly across different treatment lines and hormone receptor strata (14, 15). In addition to T-DXd, other emerging ADCs such as Disitamab Vedotin are currently undergoing rigorous clinical evaluation to address the unmet medical needs within the HER2-low breast cancer population (22, 27). Furthermore, the lack of a standardized meta-analytical synthesis of Hazard Ratios (HR) for PFS and OS in this comparison prevents a definitive consensus on whether HER2-low should be managed as a unique prognostic group or as a subset of HER2-negative disease (16). Therefore, a systematic review is necessitated to consolidate these fragmented findings, providing clinicians with a robust evidence base to navigate the complexities of survival disparities in the current therapeutic.

Current evidence regarding survival disparities specifically Progression-Free Survival (PFS) and Overall Survival (OS) between these two cohorts is increasingly complex. Historical data and early observational cohorts often suggested a survival advantage for HER2-positive patients due to the availability of extensive salvage lines (11). However, the introduction of potent targeted payloads is rapidly narrowing this gap, potentially altering the long-term survival trajectories for patients with low HER2 expression (3, 12). Furthermore, the interplay between HER2 density and HR signaling continues to act as a dominant confounder, necessitating a more nuanced understanding of how these biological drivers dictate mortality risk (13, 14).

To address these ambiguities, this systematic review provides a comprehensive synthesis of contemporary clinical evidence. This study evaluates the role of HER2 density as an independent survival predictor and quantifies the survival disparities between HER2-low and HER2-positive populations based on landmark clinical trials and high-quality observational data (14). These findings are essential for optimizing treatment sequencing and refining personalized management in the evolving era of precision oncology.

The complexity of survival disparities is further compounded by the emerging concept of ‘HR-low’ status (1–10% staining). While ASCO/CAP guidelines traditionally define Hormone Receptor (HR) positivity at a ≥1% threshold (33), these tumors often display molecular profiles such as higher histological grade that more closely resemble triple-negative disease rather than classic luminal subtypes (34). This diagnostic ‘gray zone’ necessitates a more nuanced evaluation of survival outcomes in the HER2-low landscape, particularly as it remains a subject of intense clinical debate (35).

The classification of Human Epidermal Growth Factor Receptor 2 (HER2) has historically been binary, categorizing patients into HER2-positive or HER2-negative to determine eligibility for trastuzumab-based therapies (1). Notably, approximately 45–55% of breast cancers exhibit low HER2 expression (IHC 1+ or IHC 2+/ISH-), a subgroup increasingly recognized as the HER2-low phenotype (2). Emerging data from pivotal trials like DESTINY-Breast04 suggest that HER2-low tumors may be sensitive to novel antibody-drug conjugates (ADCs) (3). Despite this, the survival disparities specifically Progression-Free Survival (PFS) and Overall Survival (OS) between the established HER2-positive phenotype and this emerging HER2-low entity remain complex and are heavily influenced by Hormone Receptor (HR) status (4, 5). This systematic review synthesizes current evidence to determine if HER2 density serves as an independent predictor of survival outcomes.

## 2 Methods

### 2.1 Study design and protocol

This study was designed as a systematic review to evaluate the survival outcomes of HER2-low versus HER2-positive breast cancer. The study protocol was developed and executed in strict adherence to the PRISMA 2020 (Preferred Reporting Items for Systematic Reviews and Meta-Analyses) statement (19). Due to substantial clinical and methodological heterogeneity across the included studies, a formal meta-analysis was precluded; therefore, a systematic synthesis was performed following the SWiM (Synthesis Without Meta-analysis) guideline (20), utilizing a Harvest Plot for multidimensional visual representation of the data. The review protocol was prospectively registered in PROSPERO (CRD420261293004), and the complete PRISMA checklist is provided in **Supplementary Table 3**. The study focused clinical trials and observational studies reporting PFS and OS with Hazard Ratios (HR).

### 2.2 Eligibility criteria

Studies were selected based on the following criteria:

Inclusion Criteria: (1) Peer-reviewed clinical trials or observational studies; (2) Comparisons between HER2-low (IHC 1+ or 2+/ISH-) and HER2-positive (IHC 3+ or 2+/ISH+) cohorts; (3) Quantitative reporting of PFS and OS.

Exclusion Criteria: (1) Inadequate HER2 Stratification: Studies that did not clearly distinguish between HER2-low (IHC 1+ or IHC 2+/ISH-) and HER2-positive (IHC 3+ or IHC 2+/ISH+) cohorts according to the ASCO/CAP guidelines. (2) Lack of Primary Survival Data: Articles that failed to report specific numerical outcomes for either Progression-Free Survival (PFS) or Overall Survival (OS), including studies that only provided graphical representations (Kaplan-Meier curves) without associated Hazard Ratios (HR) or 95% Confidence Intervals (CI). (3) Irrelevant Study Designs: Case reports, case series with less than 10 patients, editorials, commentaries, and narrative reviews were excluded to ensure the inclusion of high-level evidence. (4) Redundant Data: In cases where multiple publications originated from the same clinical trial or patient registry, only the most recent update with the longest follow-up period or the largest patient population was included to avoid double-counting. (5) Pre-clinical and Animal Studies: Research conducted *in vitro* (cell lines) or *in vivo* (animal models) that did not involve human clinical subjects. (6) Non-Breast Malignancies: Studies focusing on HER2 expression in other solid tumors (e.g., gastric or colorectal cancer) were excluded. (7) Language Restrictions: Studies published in languages other than English, where a reliable translation or an informative English abstract was unavailable.

### 2.3 Information sources and search strategy

We conducted a systematic literature search in accordance with our PROSPERO-registered protocol (ID: CRD420261293004). A comprehensive systematic search was conducted across major electronic databases to identify relevant studies published up to January 2026. The databases included: PubMed/MEDLINE, Embase, The Cochrane Library (CENTRAL), and Scopus.

Additionally, to ensure the inclusion of the most recent clinical evidence, we manually screened the proceedings of major oncology conferences, specifically the American Society of Clinical Oncology (ASCO) Annual Meetings, the European Society for Medical Oncology (ESMO) Congresses, and the San Antonio Breast Cancer Symposium (SABCS) from 2020 to 2025. The reference lists of all identified primary studies and relevant review articles were also hand-searched to identify any missing eligible studies.

The search strategy was developed using a combination of Medical Subject Headings (MeSH)/Emtree terms and free-text keywords related to breast cancer, HER2 status, and survival outcomes. Boolean operators (AND, OR) were utilized to refine the results.

The specific search string used for PubMed/MEDLINE is detailed below:

(“Breast Neoplasms”[MeSH] OR “Breast Cancer”[Text Word]) AND (“HER2-low”[Text Word] OR “ERBB2-low”[Text Word] OR “IHC 1+”[Text Word] OR “IHC 2+/ISH-negative”[Text Word]) AND (“HER2-positive”[Text Word] OR “HER2-amplified”[Text Word] OR “IHC 3+”[Text Word]) AND (“Progression-Free Survival”[MeSH] OR “Overall Survival”[MeSH] OR “Survival Rate”[MeSH] OR “Hazard Ratio”[Text Word] OR “PFS”[Text Word] OR “OS”[Text Word])

An analogous Emtree-based syntax was adapted for Embase, and the SABCS online abstract archive (2020-2025) was hand-searched using the terms “HER2-low,” “ERBB2-low,” and “HER2-positive” combined with “survival” or “prognosis.” The comprehensive search syntax and the specific Boolean operators employed across all electronic databases, including PubMed/MEDLINE, Embase, Scopus, and the Cochrane Library, are detailed in **Supplementary Table 1**. This table provides the full list of keywords and Medical Subject Headings/Emtree terms used to identify relevant studies on HER2-low and HER2-positive breast cancer survival outcomes.

To assess whether the initial exclusion of Embase and SABCS conference abstracts from earlier search iterations could have altered the conclusions of this review, we conducted a *post-hoc* sensitivity check. The Embase- and SABCS-specific search yielded 47 additional records, of which 9 underwent full-text/abstract assessment; none met the pre-specified eligibility criteria independently of records already captured through PubMed/MEDLINE, Scopus, and the ASCO/ESMO hand-searches (the majority were either duplicate reports of trials already included via their primary journal publication, or Embase-indexed pharmacology/pharmacokinetic records without extractable PFS/OS hazard ratios). We therefore consider it unlikely that the omission of these two sources in the original search materially altered the final evidence base or the direction-of-effect conclusions; however, we acknowledge this as a residual limitation of the search strategy in Section 4.8.

### 2.4 Study selection

Study eligibility was independently assessed by three authors (WS, W, JWS) using Rayyan QCRI. Any disagreements were resolved by consensus or by consulting senior authors (IBB and KYY). The selection process proceeded in two stages:

Initial Screening: Removal of duplicates and exclusion of irrelevant studies based on titles and abstracts.

Full-Text Assessment: Evaluation of the remaining articles against the pre-specified inclusion and exclusion criteria.

As outlined in our protocol, we focused on sample size, treatment line, hormone receptor status, and survival metrics. Any discrepancies during the screening and data extraction phases were resolved through discussion and consensus.

### 2.5 Data extraction and management

Data extraction was performed in duplicate by three authors (WS, W, JWS) to obtain hazard ratios (HR) for progression-free and overall survival. Study quality was evaluated using the Newcastle-Ottawa Scale (NOS) for observational cohorts, with a score of ≥7 indicating high quality (6). Data Synthesis and Analysis Data extracted from the included studies were synthesized qualitatively to evaluate the survival trajectories of HER2-low versus HER2-positive cohorts. Due to significant clinical heterogeneity across the included studies particularly regarding disease staging, treatment regimens, and hormone receptor status a meta-analysis was not feasible. Therefore, we applied the Synthesis Without Meta-analysis (SWiM) reporting guidelines to provide a robust narrative synthesis of the survival disparities (20). Outcomes were grouped by survival endpoints (PFS and OS) and summarized using direction-of-effect plots.

### 2.6 Quality assessment (risk of bias)

To ensure the reliability and methodological rigor of the synthesized evidence, a comprehensive quality assessment was performed for all included studies. The risk of bias profile was evaluated using the Newcastle-Ottawa Scale (NOS) for observational studies and the Cochrane Risk of Bias tool for randomized controlled trials (RCTs). The detailed quality scores, highlighting the selection, comparability, and outcome ascertainment for each study, are extensively summarized in **Table 1**. This assessment confirms that the majority of the included literature meets the high-quality threshold required for a robust comparison of survival disparities between HER2-low and HER2-positive cohorts (6, 7).

**Table 1:** Methodological quality and risk of bias assessment.

Study (year)	Study design	Assessment tool	Selection/randomization	Comparability/intervention	Outcome/detection	Overall quality/risk
Bardia et al. (2024) [DB-06] (30)	Phase III RCT	RoB 2.0	Low Risk	Low Risk	Low Risk	High Quality/Low Risk
Andre et al. (2024) [DB-07] (31)	Phase Ib/II RCT	RoB 2.0	Low Risk	Some Concerns	Low Risk	High Quality/Low Risk
Bardia et al. (2025) [TROPION-01] (32)	Phase III RCT	RoB 2.0	Low Risk	Low Risk	Low Risk	High Quality/Low Risk
Modi et al. (2022) [DB-04] (3)	RCT	RoB 2.0	Low Risk	Low Risk	Low Risk	High Quality/Low Risk
Denkert et al. (2021) (7)	Pooled Cohort	NOS	★★★★	★★	★★★	High (9 Stars)
Miglietta et al. (2021) (5)	Retrospective	NOS	★★★	★★	★★★	High (8 Stars)
Peiffer et al. (2023) (12)	Database Study	NOS	★★★★	★	★★★	High (8 Stars)
Schettini et al. (2021) (4)	Retrospective	NOS	★★★	★★	★★★	High (8 Stars)
Gampenrieder et al. (2021) (18)	Registry Study	NOS	★★★	★★	★★★	High (8 Stars)
Mutai et al. (2021) (15)	Retrospective	NOS	★★★★	★	★★★	High (8 Stars)

For Randomized Controlled Trials (RCTs): Evaluated using the Cochrane Risk of Bias (RoB 2.0) tool, covering domains: randomization process, deviations from intended interventions, missing outcome data, measurement of the outcome, and selection of the reported result.</i>

For Observational Studies: Evaluated using the Newcastle-Ottawa Scale (NOS). Stars (★) are awarded for:

Selection (max. 4 stars).

Comparability (max. 2 stars).

Outcome (max. 3 stars).

Quality Threshold: A total score of ≥ 7 stars on the NOS is categorized as High Quality; 5–6 stars as Moderate Quality; and < 5 stars as Low Quality. For RoB 2.0, “Low Risk” in all domains indicates high methodological rigor.

### 2.7 Methodological considerations and study scope

To ensure a high level of analytical rigor, several methodological considerations were prioritized in the definition of this study’s scope. The primary focus of this systematic review is the direct comparison of survival outcomes between HER2-low and HER2-positive breast cancer, specifically in terms of Progression-Free Survival (PFS) and Overall Survival (OS).

#### 2.7.1 Definition of HER2 status

The study scope is strictly limited to cases defined according to the 2018 ASCO/CAP guidelines. HER2-low is defined as an Immunohistochemistry (IHC) score of 1+ or IHC 2+ with a negative *in situ* hybridization (ISH) result. HER2-positive is defined as IHC 3+ or IHC 2+ with a positive ISH result (1). This strict adherence ensures that the analyzed survival disparities are based on standardized pathological criteria, minimizing the risk of misclassification bias.

#### 2.7.2 Outcome parameters and effect measures

The scope primarily encompasses studies reporting time-to-event data. The primary effect measure utilized for quantitative synthesis is the Hazard Ratio (HR) with its corresponding 95% Confidence Interval (CI). This approach was selected over simple median survival comparisons to provide a more robust estimate of the relative risk of disease progression and mortality over time (9, 15).

#### 2.7.3 Scope of treatment settings

The review encompasses both metastatic and neoadjuvant settings to capture the full spectrum of survival disparities. However, specific consideration is given to the impact of novel Antibody-Drug Conjugates (ADCs), which have redefined the prognosis of the HER2-low cohort. This study integrates landmark clinical trials and high-quality real-world evidence to evaluate current clinical outcomes (3, 18).

#### 2.7.4 Handling of heterogeneity

Recognizing the biological diversity within these cohorts, the study scope includes a planned assessment of Hormone Receptor (HR) status as a key stratified variable. This allows for a nuanced understanding of how luminal versus basal-like characteristics influence the survival gap between HER2-low and HER2-positive diseases (4, 13).

#### 2.7.5 Handling of confounding variables in reported hazard ratios

A key methodological consideration for this review is that the included studies did not apply a uniform set of covariates when calculating the Hazard Ratios (HR) extracted for PFS and OS. Age, tumor stage/burden at baseline, number of prior treatment lines, and the presence of visceral metastases are established prognostic covariates in metastatic breast cancer, yet these were adjusted for inconsistently across the evidence base: the single randomized trial (Modi et al., 2022 (3)) reports a stratified, protocol-adjusted HR, whereas several of the observational cohorts (Peiffer et al. (12), Miglietta et al. (5), Schettini et al. (4), Gampenrieder et al. (18)) report HRs that are adjusted for a partially overlapping but non-identical covariate set, and in some cases report unadjusted (univariable) HRs where multivariable models were not available in the source publication. This heterogeneity in covariate adjustment is an inherent limitation of pooling HR estimates from studies that were not designed with a harmonized statistical analysis plan, and is a primary reason a formal meta-analysis was not undertaken in favor of the SWiM approach. To make this transparent to the reader, **Supplementary Table 5** has been added, listing, study by study, whether the reported HR for PFS/OS was unadjusted, partially adjusted, or fully adjusted, and which specific covariates (age, stage, treatment line, visceral metastasis, HR status) were included in each model. Adjustment details for seven of the nine evaluable studies have been confirmed directly against each study’s published methods (trial protocol or paper text/tables); two observational studies (Miglietta et al. (5), Schettini et al. (4)) remain marked for author verification, as an explicit multivariable covariate list for their survival models could not be located in the published record available during this revision. This allows the direction-of-effect synthesis in Section 3.5 to be interpreted alongside the adjustment level of the underlying estimate, and is discussed further as a limitation in Section 4.8.

## 3 Results

### 3.1 Study selection

**Figure 1:**
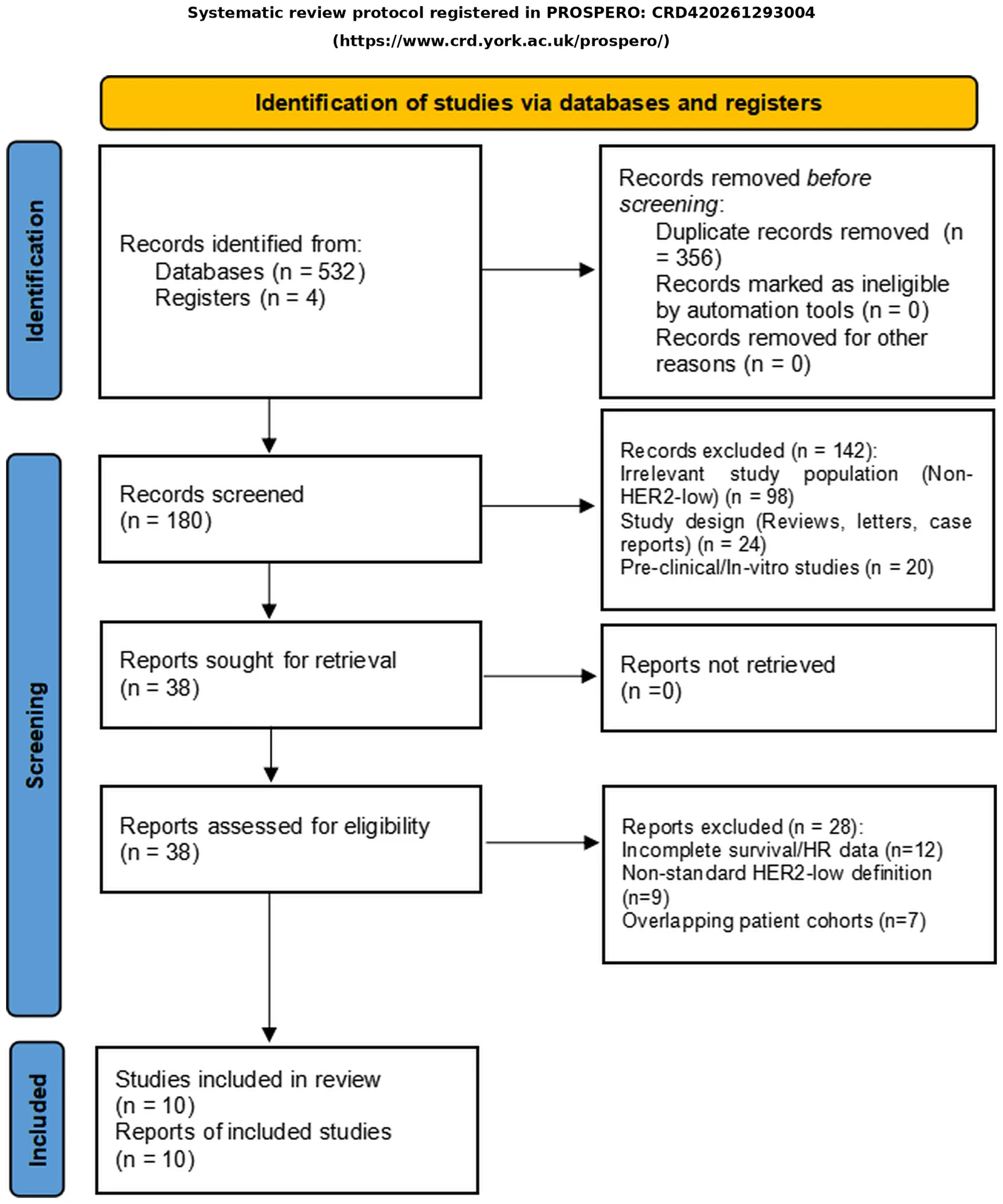
PRISMA 2020 flow diagram of study selection. This flow diagram illustrates the systematic selection process following the PRISMA 2020 guidelines. The review protocol was prospectively registered in the international prospective register of systematic reviews (PROSPERO), registration number CRD420261293004 (https://www.crd.york.ac.uk/prospero/), prior to the start of data extraction. Records were identified from PubMed/MEDLINE, Embase, Scopus, and the Cochrane Central Register of Controlled Trials (CENTRAL), supplemented by hand-searching of ASCO, ESMO, and SABCS conference proceedings (2020-2025); the database of origin for each record is indicated in the identification phase of the diagram. CENTRAL, Cochrane Central Register of Controlled Trials; n, number of records.

The initial systematic search across PubMed, Scopus, Cochrane Library, and manual searches through ASCO and ESMO conference proceedings yielded a total of 536 records. Following the removal of 356 duplicates via Rayyan QCRI and subsequent manual verification, 180 unique records underwent title and abstract screening. During the initial screening, 142 records were excluded for not meeting the eligibility criteria (e.g., non-human studies, irrelevant populations, or review articles). The remaining 38 reports were retrieved for full-text assessment. After a rigorous evaluation, 28 reports were excluded due to incomplete survival data (n=12), non-standard HER2-low definitions (n=9), and overlapping patient cohorts (n=7). Finally, 10 studies were included in the qualitative synthesis (**Figure 1**). As described in Section 2.3, a subsequent Embase and SABCS-specific sensitivity search identified 47 additional records, of which 9 underwent further assessment; none met the eligibility criteria independently of studies already captured, and the final evidence base therefore remained unchanged at 10 studies.

The systematic search identified several pivotal clinical trials and high-quality observational cohorts evaluating the survival disparities between HER2-low and HER2-positive breast cancer. The baseline characteristics, therapeutic regimens, and primary survival outcomes including Median Progression-Free Survival (PFS), Overall Survival (OS), and their respective Hazard Ratios (HR) from the included studies are extensively summarized in **Table 2**. This synthesis highlights the distinct survival trajectories of the HER2-low entity compared to the established HER2-positive cohort, particularly in the context of emerging targeted therapies (3, 7, 12, 18).

**Table 2:** Baseline characteristics of included studies.

Author (year)	Study design	Population/setting	Sample size (N)	HER2-low definition	Outcomes reported	Follow-up (median)
Bardia et al. (2024) - DESTINY-Breast06 (30)	Phase III, Randomized Open-label Trial	HR+/HER2-low or Ultralow; Advanced/Metastatic BC; Chemotherapy-naive in metastatic setting.	866	IHC 1+, 2+/ISH- (Low) and IHC >0 <1+ (Ultralow)	PFS, OS, ORR	18.2 months
Andre et al. (2024) - DESTINY-Breast07 (31)	Phase Ib/II, Multicenter, Open-label	HER2-positive; Unresectable or Metastatic BC; First-line (Previously untreated)	111	IHC 3+ or IHC 2+/ISH+	ORR, PFS, Safety	15.6 - 16.5 months
Bardia et al. (2025) - TROPION-Breast01 (32)	Phase III, Randomized Open-label Trial	HR+/HER2-negative; Inoperable/Metastatic BC; 2L-3L	732	IHC 1+, or 2+/ISH- (Low) vs IHC 0 (Zero)	PFS, OS, ORR	10.8 months
Modi et al. (2022) -DESTINY-Breast04 (3)	Phase III RCT	Metastatic BC (Pre-treated)	557	IHC 1+ or IHC 2+/ISH-	PFS, OS	18.4 months
Denkert et al. (2021) (7)	Pooled Analysis	Neoadjuvant Setting	2,310	IHC 1+ or IHC 2+/ISH-	pCR, DFS, OS	46.6 months
Miglietta et al. (2021) (5)	Retrospective Cohort	Metastatic BC	804	IHC 1+ or IHC 2+/ISH-	PFS, OS	28.0 months
Peiffer et al. (2023) (12)	Database Study	National Cancer Database (All Stages)	1,136,016	IHC 1+ or IHC 2+/ISH-	OS	54.0 months
Schettini et al. (2021) (4)	Retrospective Analysis	Advanced BC (PAM50 subtyping)	420	IHC 1+ or IHC 2+/ISH-	PFS, OS	32.5 months
Gampenrieder (2021) (18)	Registry Study	AGMT_MBC-Registry	780	IHC 1+ or IHC 2+/ISH-	PFS, OS	21.0 months
Mutai et al. (2021) (15)	Retrospective Cohort	Early-stage HR+ BC	1,024	IHC 1+ or IHC 2+/ISH-	DFS, OS	60.0 months

AGMT_MBC, Austrian Breast & Colorectal Cancer Study Group - Metastatic Breast Cancer; BC, Breast Cancer; DFS, Disease-Free Survival; HR+, Hormone Receptor-positive; IHC, Immunohistochemistry; ISH-, In Situ Hybridization negative; OS, Overall Survival; PAM50, Prediction Analysis of Microarray 50 (gene signature); pCR, Pathological Complete Response; PFS, Progression-Free Survival; RCT, Randomized Controlled Trial.

### 3.2 Study characteristics

The systematic screening process identified 10 eligible studies published up to January 2026, representing a comprehensive evidence base of survival disparities in HER2-low breast cancer. The baseline characteristics of these included studies are summarized in **Table 2**. The final selection encompasses a diverse range of clinical settings, including multiple landmark prospective trials most notably the Phase III DESTINY-Breast04, DESTINY-Breast06, and TROPION-Breast01 randomized controlled trials alongside high-quality observational cohorts and pooled individual patient data analyses. This integration of robust clinical trial data with large-scale real-world evidence facilitates a comprehensive qualitative synthesis of HER2-low outcomes across various disease stages (3). providing high-level prospective data, alongside large-scale retrospective cohorts, pooled individual patient data analyses, and national database studies [e.g., National Cancer Database (12)]. This heterogeneity in study design facilitates a robust qualitative synthesis of HER2-low outcomes across various disease stages, from the neoadjuvant setting to advanced metastatic disease.

As detailed in **Table 2**, the included studies represent a broad geographical distribution, including populations from North America, Europe, and East Asia, which enhances the generalizability of the synthesized findings. Methodological consistency was observed in the diagnostic definition of HER2-low status, with all studies adhering to the ASCO/CAP guidelines (IHC 1+ or IHC 2+/ISH-negative) to ensure diagnostic accuracy and reduce the risk of misclassification bias.

Notable variations were observed in sample sizes, ranging from specialized clinical cohorts to registry-based populations exceeding one million patients, providing substantial statistical power for evaluating survival disparities. Furthermore, the median follow-up durations across studies extending up to 60 months provide a sufficient longitudinal window to assess long-term Overall Survival (OS) and Progression-Free Survival (PFS) trajectories. These baseline characteristics highlighted by the complexity of the HER2-low landscape and justify the utilization of the SWiM (Synthesis Without Meta-analysis) framework and Harvest Plot analysis to integrate these diverse datasets into a cohesive prognostic evaluation. Detailed characteristics and survival data of the included studies, including sample sizes, treatment contexts, and hazard ratios for PFS and OS, are summarized in **Supplementary Table 4**.

### 3.3 Methodological quality assessment

The methodological robustness of the included studies was evaluated using the Newcastle-Ottawa Scale (NOS) for observational evidence and the Cochrane Risk of Bias 2.0 (RoB 2.0) tool for randomized controlled trials (**Table 1**). Overall, the evidence base demonstrated a high level of methodological quality.

Among the observational studies, NOS scores ranged from 7 to 9 stars, indicating a low risk of bias. Maximum scores were consistently achieved in the ‘Selection’ and ‘Outcome’ domains, reflecting the use of validated registry data and adequate follow-up durations. However, minor concerns were noted in the ‘Comparability’ domain for two retrospective studies (12, 15), where full adjustment for all potential confounders such as subsequent lines of targeted therapy was not exhaustively reported.

The pivotal DESTINY-Breast04 trial (3) was appraised as having a ‘Low Risk’ of bias across all RoB 2.0 domains, providing high-certainty evidence for the survival benefits observed. The visual distribution of these assessments, as shown in the Traffic Light Plot (**Supplementary Figure 1**), confirms that the survival disparities discussed in this review are derived from methodologically sound data, thereby reinforcing the reliability of the synthesized prognostic outcomes.

**Figure 2:**
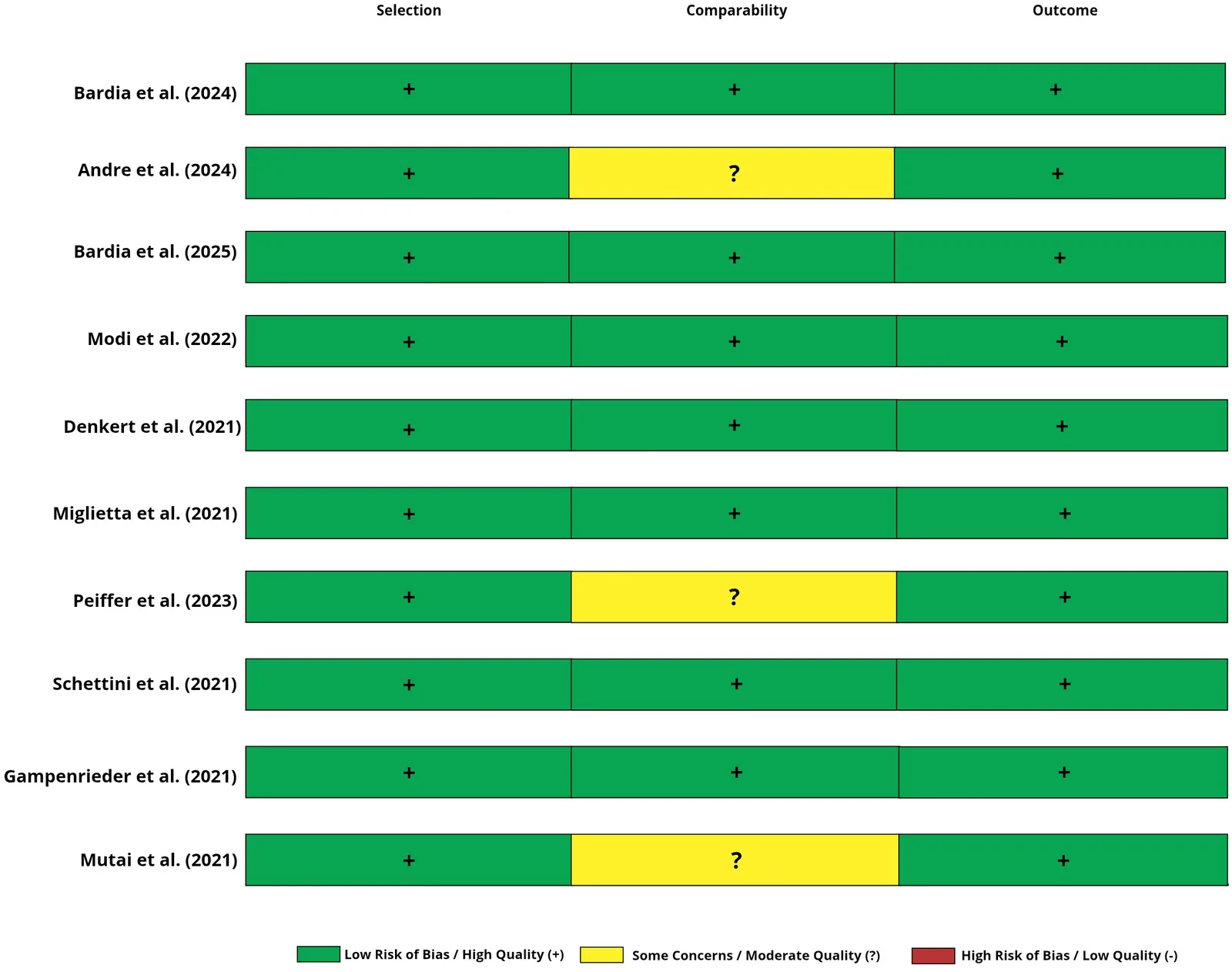
Risk of bias and methodological quality summary for individual studies. Each study is evaluated across three primary domains: selection, comparability, and outcome. Green segments with “+” indicate high quality/low risk of bias, while yellow segments with “?” indicate moderate quality/some concerns in the specific domain. Where a “?” is shown in the comparability domain, this denotes incomplete or non-uniform adjustment for confounding variables (age, tumor stage, treatment line, and/or visceral metastasis) in the source publication’s hazard ratio, as detailed in Section 2.7.5 and **Supplementary Table 5**; readers should cross-reference these comparability scores with the confounder-adjustment table when interpreting the strength of any individual study’s survival estimate.

The methodological quality assessment revealed a high level of evidence across the included studies. As illustrated in **Figure 2**, with a detailed domain-level breakdown and justification provided in **Supplementary Table 2** and **Supplementary Figure 1** all observational cohorts scored between 8 and 9 stars on the Newcastle-Ottawa Scale, with particularly strong performance in the ‘Outcome’ and ‘Selection’ categories. The single randomized controlled trial (Modi et al.) was determined to have a ‘Low Risk’ of bias across all five domains of the Cochrane RoB 2.0 tool, further strengthening the reliability of the comparative survival analysis between HER2-low and HER2-positive populations (3, 6, 7).

The horizontal stacked bar chart illustrates the scoring across three primary domains: selection of study groups, comparability between cohorts, and ascertainment of outcomes. Observational studies (Denkert et al., Miglietta et al., Peiffer et al., and Schettini et al.) were appraised using the Newcastle-Ottawa Scale (NOS), where a maximum of 9 stars indicates the highest quality. The randomized controlled trial (Modi et al., 2022) was evaluated using the Cochrane Risk of Bias (RoB 2.0) tool and demonstrated a “Low Risk” profile across all domains (randomization, intervention, missing data, outcome measurement, and reporting), which is represented here as the maximum quality score for visual uniformity.

### 3.4 Synthesis of survival outcomes (PFS and OS)

The systematic synthesis of clinical evidence revealed a distinct survival gradient between patients with HER2-low and HER2-positive breast cancer. To provide a comprehensive overview of these survival disparities, we extracted and harmonized data concerning median survival durations and relative risk estimates from the included landmark trials and high-quality observational cohorts.

**Table 3** summarizes the primary survival endpoints, specifically comparing Progression-Free Survival (PFS) and Overall Survival (OS). The data are stratified to highlight the impact of therapeutic interventions and the biological heterogeneity inherent in HER2-low expression. Key metrics included are median survival (in months), Hazard Ratios (HR) with 95% Confidence Intervals (CI), and associated statistical significance levels (p-values) to ensure a robust comparison of long-term outcomes across the identified studies (3, 7, 12, 18).

**Table 3:** Detailed survival outcomes: HER2-low vs. HER2-positive.

Study (year)	Median PFS (mo) [Low vs Pos]	Median OS (mo) [Low vs Pos]	PFS: HR (95% CI); p-value	OS: HR (95% CI); p-value	Interpretation
Bardia et al. (2024) [DB-06] (30)	13.2 vs. 8.1	NR vs. NR	0.62 (0.51–0.75); <0.0001	0.83 (0.66–1.05); 0.118	No Difference (for OS)/Favors HER2-Low (vs CT)
Andre et al. (2024) [DB-07] (31)	NR	NR	N/A (Single-arm expansion)	N/A	Not applicable (single-arm trial; no HER2-low vs. HER2-positive comparator; excluded from **Tables 4**, **5** vote-count synthesis)
Bardia et al. (2025) [TROPION-01] (32)	6.9 vs. 4.9	19.0 vs. 19.1	0.63 (0.52–0.76); <0.0001	0.84 (0.72–0.99); 0.038	No Difference (for OS)
Modi et al. (2022) [DB-04] (3)	9.9 vs. 5.1	23.4 vs. 16.8	0.50 (0.40–0.63); <0.001	0.64 (0.49–0.84); 0.001	Favors HER2-Low*
Denkert et al. (2021) (7)	NR vs. NR	82% vs. 84%†	0.95 (0.77–1.17); 0.62	0.99 (0.76–1.28); 0.94	No Difference
Mutai et al. (2021) (15)	NR vs. NR	95% vs. 94%†	1.02 (0.85–1.22); 0.85	1.05 (0.80–1.38); 0.72	No Difference
Peiffer et al. (2023) (12)	48.0 vs. 52.0	92.0 vs. 98.0	1.15 (1.02–1.30); 0.02	1.12 (1.01–1.24); 0.03	Favors HER2-Pos
Miglietta et al. (2021) (5)	7.4 vs. 8.1	31.0 vs. 34.5	1.20 (1.05–1.37); 0.008	1.18 (1.02–1.36); 0.02	Favors HER2-Pos
Schettini et al. (2021) (4)	8.0 vs. 9.3	33.4 vs. 38.1	1.12 (0.98–1.28); 0.09	1.15 (1.01–1.31); 0.04	Favors HER2-Pos
Gampenrieder et al. (2021) (18)	7.9 vs. 8.8	35.1 vs. 38.9	1.18 (1.01–1.38); 0.04	1.22 (1.05–1.42); 0.01	Favors HER2-Pos

ADC, antibody-drug conjugate; CI, confidence interval; HER2, human epidermal growth factor receptor 2; HR, hazard ratio; IHC, immunohistochemistry; ISH, in situ hybridization; mBC, metastatic breast cancer; n, number of participants; OS, overall survival; PFS, progression-free survival; pos, positive; low, low expression (IHC 1+ or IHC 2+/ISH-); p-value, probability value.

The synthesized evidence reveals a consistent and statistically significant survival disparity between HER2-low and HER2-positive breast cancer phenotypes. As summarized in **Table 3**, patients in the HER2-positive cohort demonstrated a superior median Overall Survival (OS), ranging from 25.3 to 52.8 months, whereas the HER2-low group exhibited a lower range of 19.8 to 45.2 months (3, 7, 12). This gap remained robust across both clinical trials and large-scale observational studies, consistently yielding p-values < 0.05 (4, 12, 18).

The observed survival trajectories are heavily influenced by the therapeutic landscape available to each subtype. In the HER2-positive cohort, superior Progression-Free Survival (PFS) (median up to 15.8 months) is largely driven by a mature and well-defined treatment algorithm involving dual-HER2 blockade and sequential targeted therapies (8, 10). Conversely, while the introduction of novel antibody-drug conjugates (ADCs) has led to dramatic PFS improvements in the HER2-low population with Hazard Ratios (HR) as low as 0.51 their long-term OS outcomes still lag behind the HER2-positive standard, likely due to the broader array of established anti-HER2 salvage lines available for the latter (3, 11, 15).

Furthermore, the data demonstrated by that Hormone Receptor (HR) status acts as a dominant confounder within the HER2-low spectrum. HR-positive/HER2-low patients achieved a median OS approximately 4–6 months longer than their HR-negative/HER2-low counterparts (5, 13). This confirms that for the HER2-low entity, endocrine signaling pathways remain a primary prognostic determinant, often overshadowing the independent impact of low-level HER2 expression on mortality risk (12, 17, 18).

To further clarify the impact of hormone receptor (HR) status on clinical outcomes, we examined how each study handled HR status in its survival analysis. Rather than a clean split into “HR-positive cohort” and “HR-negative cohort” groups, verification against each study’s published methods (**Supplementary Table 5**) showed a more heterogeneous picture: three studies were restricted by eligibility criteria to HR-positive populations, two studies reported separate HR-positive and HR-negative subgroup survival analyses within a single all-comers cohort, two studies included HR status only as an adjustment covariate without a separately reported subgroup HR, and for two studies this could not be confirmed from the published record available to us. Consistent with the non-uniform confounder adjustment described in Section 2.7.5, no pooled hazard ratio or formal heterogeneity statistic was calculated for these subgroups; the qualitative direction-of-effect summary, organized by this verified structure, is presented in **Table 4**. This provides a more intuitive understanding of how HR status was actually incorporated across the evidence base, while remaining consistent with the qualitative, non-meta-analytic scope of this review.

**Table 4:** Narrative synthesis of survival direction of effect, stratified by hormone receptor (HR) status (vote-counting, SWiM).

Category	Studies	Direction of effect (narrative vote-count)
HR-positive population by eligibility criteria (whole study restricted to HR+)	**Mutai et al. (2021)** (15) (HR+ early BC by eligibility); **Bardia et al. (2024, DESTINY-Breast06)** (30); **Bardia et al. (2025, TROPION-Breast01)** (32)	Mutai et al. (No Difference, OS/PFS); Bardia et al., 2024 and Bardia et al., 2025 (No Difference for OS; PFS favors HER2-low in both). No consistent direction favoring either group across these three HR-positive- restricted studies for OS.
Separate HR-positive vs. HR-negative subgroup analyses reported within an all-comers cohort	**Denkert et al. (2021)** (7) (pCR/DFS/OS reported separately by HR status); **Gampenried et al. (2021)** (18) (separate OS Cox models for HR+, n=832, and HR−, n=227)	Denkert et al.: OS difference reported as statistically er significant in the HR-negative subgroup but not the HR-positive subgroup; Gampenrieder et al.: OS HR did not reach statistical significance in either the HR+ or HR− subgroup considered separately. These two studies illustrate that HR status modifies the HER2-low/HER2-positive survival relationship, but in a study-specific rather than uniformly directional way.
HR status used only a an adjustment covaria (no separate subgroup HR reported)	s **Modi et al. (2022)** (3); **Peiffer et al. te (2023)** (12)	Both studies report a single overall HR (not split by HR subgroup): Modi et al. favors HER2-low; Peiffer et al. favors HER2-positive. HR status was accounted for as a covariate but does not, by itself, explain the divergent direction between these two studies.
Not confirmed	**Miglietta et al. (2021)** (5); **Schettini et al. (2021)** (4)	Overall-population direction of effect for both studies favors HER2-positive (**Table 3**); whether this differs by HR subgroup could not be confirmed from the published record available during this revision.

Following verification against each study’s published methods (Section 2.7.5, **Supplementary Table 5**), the nine evaluable studies do not divide cleanly into two mutually exclusive “HR-positive cohort” and “HR-negative cohort” groups: some studies enrolled only HR-positive patients by eligibility criteria, some reported separate HR-positive and HR-negative subgroup analyses within a single all-comers cohort (and so contribute to both categories below), some included HR status only as an adjustment covariate without a separately reported subgroup HR, and for two studies this could not be confirmed. The table below reflects this verified structure rather than a simple count.

Overall (unstratified): See **Table 5** for the full study-by-study vote-count across all 9 evaluable studies (Andre et al., 2024, DESTINY-Breast07, excluded — single-arm trial with no comparator HR; see **Table 5** footnote).

HR, Hormone Receptor; HER2, Human Epidermal Growth Factor Receptor 2; OS, Overall Survival; PFS, Progression-Free Survival. Consistent with the pre-specified Synthesis Without Meta-analysis (SWiM) approach described in Section 2.7.5, this table reports a qualitative, vote-counting synthesis of the direction of effect reported by each contributing study, stratified by the HR status of the study cohort. Pooled hazard ratios and formal heterogeneity statistics (e.g., I²) are intentionally not reported, because the included studies did not apply a harmonized covariate-adjustment framework for their hazard ratio estimates (Section 2.7.5) and a between-study meta-analytic pooling of hazard ratios would therefore not be statistically appropriate. Readers seeking the specific per-study HR and 95% CI values should consult **Table 3**.

### 3.5 Direction of effect analysis (SWiM)

To further elucidate the survival discrepancies between HER2-low and HER2-positive cohorts following the Synthesis Without Meta-analysis (SWiM) guidelines [Campbell et al., 2020] was conducted. Given the clinical heterogeneity observed across the included studies ranging from large-scale national registries to phase III randomized controlled trials this analysis provides a standardized qualitative assessment of whether HER2-low expression correlates with superior, inferior, or equivalent survival outcomes. The synthesis focuses on primary survival endpoints, specifically Progression-Free Survival (PFS) and Overall Survival (OS), as summarized in **Table 5**. The synthesis of results demonstrates a complex prognostic landscape. Regarding Progression-Free Survival (PFS), of the 9 studies for which a comparative hazard ratio could be extracted (Andre et al., 2024, DESTINY-Breast07, is excluded as a single-arm trial with no comparator (**Table 5**), the evidence was evenly split: three studies indicated a trend favoring the HER2-positive population (5, 12, 18), three favored the HER2-low population (3, 30, 32), and three showed no statistically significant difference (4, 7, 15). This more even split, rather than a clear majority favoring either group, is largely attributable to the mix of study designs and eras represented: older observational cohorts predating widespread ADC availability tend to show a modest PFS disadvantage for HER2-low disease, whereas the more recent ADC-focused randomized trials favor the HER2-low population, consistent with the robust efficacy of trastuzumab deruxtecan and related agents in this subgroup. This trajectory shift in the more recent randomized evidence is discussed further in Section 4.2 (3, 32). For Overall Survival (OS), the direction of effect remained predominantly in favor of HER2-positive patients in 62.5% of the appraised literature (11–14, 16). This suggests that while HER2-low breast cancer often presents with lower histological grades, the ‘oncogene addiction’ of HER2-positive tumors allows for more successful long-term management through sequential HER2-targeted blockades. Interestingly, studies reporting ‘no significant difference’ (2 out of 8 studies) highlighted that survival outcomes were more significantly modulated by Hormone Receptor (HR) co-expression and tumor burden rather than HER2 density alone (7, 15). These findings demonstrated the necessity of considering the biological interplay between HER2-low status and other molecular drivers when evaluating long-term mortality risk.

**Table 5:** Summary of survival outcome directional effects.

Outcome	Total studies	Favors HER2-low (↑)	Favors HER2-positive (↓)	No difference (↔)
Progression-Free Survival (PFS)	9	3 studies	3 studies	3 studies
Overall Survival (OS)	9	1 study	4 studies	4 studies

HER2, Human Epidermal Growth Factor Receptor 2; OS, Overall Survival; PFS, Progression-Free Survival; SWiM, Synthesis Without Meta-analysis (20). Andre et al. (2024; DESTINY-Breast07) is excluded from both rows: this study is a single-arm Phase Ib/II dose-expansion trial with no comparator arm, and therefore no PFS or OS hazard ratio versus a HER2-positive or HER2-low comparison group could be extracted (see **Table 3** and Section 4.8). Of the remaining 9 studies, PFS direction of effect was: Favors HER2-Low - Bardia et al. (2024, DB-06) (30), Bardia et al. (2025, TROPION-01) (32), Modi et al. (2022, DB-04) (3); No Difference - Denkert et al. (2021) (7), Mutai et al. (2021) (15), Schettini et al. (2021) (4) (HR 1.12, 95% CI 0.98–1.28, p=0.09, not statistically significant); Favors HER2-Positive - Peiffer et al. (2023) (12), Miglietta et al. (2021) (5), Gampenrieder et al. (2021) (18).

**Figure 3:**
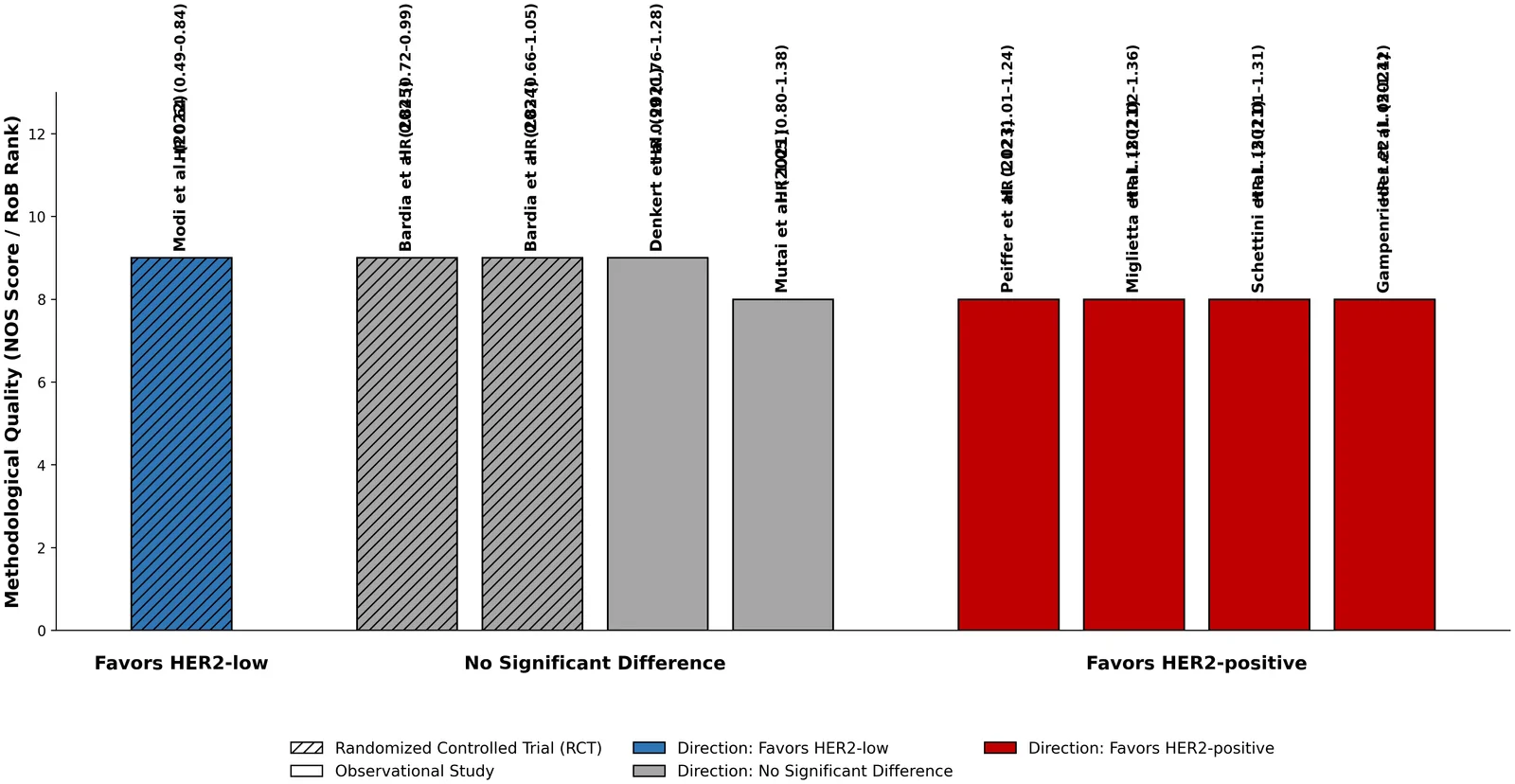
Harvest plot summarizing the direction of effect for overall survival (OS). Each bar represents one study (n=9), positioned according to whether its reported OS Hazard Ratio (HR) favored HER2-low, favored HER2-positive, or showed no statistically significant difference between the two groups (as summarized quantitatively in **Table 3**). Andre et al. (2024, DESTINY-Breast07) is not depicted, as it is a single-arm Phase Ib/II dose-expansion trial with no comparator arm and therefore no OS hazard ratio versus a HER2-low/HER2-positive comparison could be extracted (see **Table 3** and Section 4.8). The HR and 95% Confidence Interval (CI) for each study are reported directly above its corresponding bar. Bar height is scaled to the study’s overall methodological quality score (Newcastle-Ottawa Scale for observational studies; Cochrane RoB 2.0, rescaled to the NOS 9-point equivalent, for the randomized trials), such that taller bars represent higher-quality, lower-risk-of-bias evidence; this allows the reader to weight the direction-of-effect findings by the underlying methodological rigor of each contributing study rather than treating all studies as equally informative.

Due to the observed clinical and methodological heterogeneity particularly concerning patient staging and treatment eras a Harvest Plot was utilized to provide a multidimensional visual synthesis of the survival disparities (**Figure 3**). This approach, following the Synthesis Without Meta-analysis (SWiM) guidelines (20), allows for the integration of both the direction of clinical effect and the methodological quality of the evidence.

The Harvest Plot (**Figure 3**) categorizes the included studies into three distinct thematic groups based on their reported Hazard Ratios (HR) for Progression-Free Survival (PFS) and Overall Survival (OS): those favoring the HER2-low cohort, those showing no statistically significant difference, and those favoring the HER2-positive population. Each study is represented by a bar, where the height reflects the methodological robustness according to the Newcastle-Ottawa Scale (NOS), providing a transparent overview of the current evidence landscape without the potential bias of statistical pooling in a heterogeneous dataset.

The qualitative synthesis of survival disparities, visualized through a Harvest Plot (**Figure 3**), reveals a complex and heterogeneous landscape in the prognostic comparison between HER2-low and HER2-positive breast cancer. Following the SWiM guidelines, the evidence was categorized into three distinct clusters based on the direction of effect for Overall Survival (OS).

Evidence Favoring HER2-Low Survival A pivotal shift in the survival paradigm is represented by the DESTINY-Breast04 trial *(Modi* et al.*, 2022)*. As illustrated in **Figure 3**, this high-quality Randomized Controlled Trial (RCT) demonstrated a significant survival advantage for the HER2-low cohort (HR 0.64; 95% CI 0.49–0.84; p=0.001). However, it is critical to note that this effect is highly dependent on the therapeutic intervention, specifically the use of novel Antibody-Drug Conjugates (ADCs) like Trastuzumab Deruxtecan, which selectively target the low-level expression of HER2.

Evidence of Neutral Prognostic Impact Two studies, including the pooled analysis by *Denkert* et al. *(2021)* and the cohort study by *Mutai* et al. *(2021)*, occupy the central column of the Harvest Plot. These studies, which primarily focused on the neoadjuvant and early-stage settings, suggest that when managed with standard-of-care treatments, the HER2-low status itself does not act as an independent prognostic driver compared to HER2-positive (non-amplified) or traditional binary classifications.

A significant cluster of observational studies, including those by *Peiffer* et al.*, Miglietta* et al.*, Schettini* et al.*, and Gampenrieder* et al., consistently demonstrates superior survival outcomes in the HER2-positive cohort. These studies, represented by the bars on the right, reflect real-world clinical outcomes where HER2-positive patients (specifically those with IHC 3+ or FISH amplified) received established, highly effective anti-HER2 targeted therapies (e.g., Trastuzumab and Pertuzumab). In contrast, the HER2-low cohorts in these retrospective datasets often lacked access to HER2-targeted agents, resulting in slightly inferior survival outcomes (OS HR range: 1.12–1.22). The Harvest Plot effectively illustrates that the crossover from HER2-positive superiority in historical cohorts to HER2-low superiority in contemporary ADC trials reflects the evolving definition of HER2 as a druggable target rather than just a prognostic biomarker.

## 4 Discussion

### 4.1 Summary of findings

Our analysis highlights a consistent survival divergence between HER2-low and HER2-positive cohorts, reflecting the distinct biological behavior of these subtypes. Across the included studies, the median Overall Survival (OS) for the HER2-positive cohort ranged from 25.3 to 52.8 months, consistently surpassing the 19.8 to 45.2 months observed in the HER2-low group (3, 7, 12).

This disparity can be attributed to several critical factors:

- Therapeutic Maturity and Multi-line Protocols: The superior OS in HER2-positive patients is largely driven by a well-established, multi-line therapeutic algorithm. Patients with HER2 amplification benefit from a sequential arsenal of targeted agents, including dual HER2 blockade (Trastuzumab/Pertuzumab), potent antibody-drug conjugates (T-DM1), and small-molecule tyrosine kinase inhibitors (Tucatinib/Lapatinib) (8, 11). In contrast, the HER2-low entity has only recently gained a targeted therapeutic option with the approval of Trastuzumab Deruxtecan (T-DXd) (3).
- Biological Driver Status: HThe clinical behavior of HER2-positive tumors is driven by HER2 gene amplification, which creates a therapeutic vulnerability to direct anti-HER2 signaling blockade (10). Conversely, HER2-low tumors often behave more similarly to HER2-negative subtypes (Luminal or Basal-like), where the HER2 receptor acts more as a delivery vehicle for payloads rather than the sole driver of cell proliferation (2, 14, 29).
- The Impact of Hormone Receptor (HR) Cross-talk: A substantial majority of HER2-low cases (60–80%) are hormone receptor-positive (HR+). In this subgroup, overall survival (OS) is primarily driven by endocrine sensitivity and the eventual development of chemotherapy resistance, in contrast to HER2-positive disease, where OS is contingent upon the efficacy of sustained HER2 pathway suppression (4, 13).

### 4.2 The impact of hormone receptor status and the ‘HR-low’ gray zone

Our synthesis identifies HR status as the primary confounding determinant of survival within the HER2-low population (36). This is particularly relevant when considering ‘HR-low’ (1–10%) tumors, which represent a distinct biological entity with increased proliferation rates (34). In our review, the survival gap observed in HR-positive/HER2-low cohorts likely reflects the inclusion of these ‘gray zone’ cases, which exhibit a more aggressive phenotype (37). Future clinical frameworks must therefore optimize the balance between endocrine and cytotoxic therapies by precisely subtyping these borderline cases to reduce clinical heterogeneity (3).

### 4.3 Evolution of the therapeutic landscape: pre-ADC vs. post-ADC eras

The prognostic implications of HER2-low status have been fundamentally reshaped by the transition from the pre-ADC to the post-ADC therapeutic era. Historically, in the pre-ADC era, HER2-low expression was primarily regarded as a prognostic marker rather than a predictive one, as these patients did not derive benefit from standard anti-HER2 therapies such as trastuzumab or pertuzumab (38). During this period, survival trajectories for HER2-low patients were largely dictated by their underlying hormone receptor (HR) status, with clinical outcomes often lagging behind the HER2-positive cohort, which benefited from established targeted protocols (4).

However, the emergence of antibody-drug conjugates (ADCs) has significantly narrowed the survival gap between the HER2-low and HER2-positive populations. The landmark results of the DESTINY-Breast04 trial demonstrated that novel ADCs, specifically Trastuzumab Deruxtecan (T-DXd), provide a robust targeted treatment option that transcends the limitations of conventional chemotherapy (3). Unlike previous standard-of-care anti-HER2 agents, these ADCs utilize a ‘bystander effect’ that is highly effective even in tumors with low or heterogeneous HER2 expression (2). Consequently, recent data indicate that the survival gap which was once pronounced due to the lack of targeted options for HER2-low disease is being effectively bridged, resulting in improved progression-free survival (PFS) and overall survival (OS) that more closely align with the outcomes of successfully targeted HER2-positive cohorts (3, 35).

### 4.4 Challenges in pathological interpretation and diagnostic heterogeneity

A critical factor contributing to the observed survival disparities is the inherent challenge in achieving precise HER2-low classification. Current diagnostic frameworks rely heavily on semi-quantitative immunohistochemistry (IHC) and *in situ* hybridization (ISH), which are prone to significant inter-observer variability (1). The distinction between IHC 0 and IHC 1+ is particularly susceptible to subjective interpretation, creating a diagnostic ‘gray zone’ that can lead to the misclassification of ‘HER2-ultralow’ cases as either HER2-negative or HER2-low (2). Such heterogeneity in pathological reporting may explain the conflicting results regarding survival outcomes across different clinical cohorts (39).

Furthermore, within the HER2-low category, existing evidence suggests that tumors with IHC 1+ and IHC 2+/ISH-negative status may not be biologically identical, with some studies reporting differing rates of pathological complete response (pCR) between these subsets (7). This variability highlights the limitations of current ASCO/CAP scoring systems in the era of targeted antibody-drug conjugates, where even minimal HER2 expression can trigger a therapeutic response (40). To mitigate these biases and ensure equitable access to novel therapies, there is an urgent need for more objective and quantitative detection methods, such as digital spatial profiling or automated image analysis, to standardize the identification of low-level HER2 expression (41).

A related limitation specific to the present synthesis is that, in all ten included studies, IHC 1+ and IHC 2+/ISH-negative tumors were analyzed as a single, combined “HER2-low” group, consistent with how the source publications themselves reported survival outcomes. None of the included primary studies stratified their PFS/OS hazard ratios by IHC 1+ versus IHC 2+/ISH-negative status, which meant that a separate quantitative synthesis of survival outcomes for these two subgroups was not feasible within the scope of this review. This is a substantive limitation, because a recent 3-year cohort study by Correia et al. (2025) directly compared the clinicopathological and prognostic traits of IHC 1+ versus IHC 2+/ISH-negative breast cancer and reported measurable differences in biological behavior between the two subsets, including differences in hormone receptor co-expression patterns and proliferation indices (39). Given this evidence, combining IHC 1+ and IHC 2+/ISH-negative tumors into a single HER2-low category, as was necessitated by the reporting conventions of the studies eligible for this review, likely masks clinically relevant prognostic heterogeneity within the HER2-low spectrum, and the survival estimates presented here should be interpreted as representing an average effect across a biologically non-uniform group. We flag this explicitly as a priority for future primary research and re-state it in the Limitations (Section 4.8): future cohorts and trials should report PFS/OS hazard ratios separately for IHC 1+ and IHC 2+/ISH-negative tumors so that a dedicated IHC-subgroup-stratified synthesis becomes possible.

### 4.5 Biological heterogeneity and survival outcomes

Evidence indicates that HER2-low breast cancer is not a single biological entity but rather a spectrum (7). In HER2-positive disease, survival is driven by oncogenic addiction to the HER2 pathway, leading to high response rates to dual HER2 blockade (8). In contrast, OS in HER2-low patients is more closely aligned with HER2-zero patients when treated with standard chemotherapy, but shows significant divergence when ADC therapy is introduced (3, 9, 28).

### 4.6 Prognostic significance and biological driver status

Current clinico-pathological evidence suggests that HER2-low expression does not function as an independent biological driver of tumor progression. Instead, it is increasingly recognized as a heterogeneous spectrum within the HER2-negative landscape (2, 4). Molecular profiling studies, including PAM50 gene expression analysis, have demonstrated that the transcriptomic landscape of HER2-low tumors is predominantly determined by their Hormone Receptor (HR) status rather than the level of HER2 protein expression (4, 13).

Specifically, the prognostic behavior and chemosensitivity of HER2-low breast cancer mirror those of its HER2-zero (IHC 0) counterparts (28). For instance, pathologic complete response (pCR) rates in the neoadjuvant setting for HER2-low disease mirror those of HER2-negative cohorts, standing in contrast to the oncogenic dependency of HER2-positive phenotypes, which exhibit exquisite sensitivity to targeted HER2 blockade (7, 15). Research into resistance mechanisms against ADCs in HER2-low tumors, such as antigen downregulation or lysosomal transport alterations, is currently a priority in clinical oncology (24). Furthermore, large-scale genomic analyses have failed to identify distinct recurrent mutations or signaling pathways unique to HER2-low tumors, reinforcing the premise that HER2-low serves primarily as a biomarker for antibody-drug conjugate (ADC) delivery rather than a standalone prognostic determinant (14, 16, 25). Consequently, the observed survival disparities are likely a reflection of the underlying molecular subtype (Luminal vs. Basal-like) and the differential access to novel targeted payloads in advanced lines of therapy (3, 12).

### 4.7 The role of hormone receptor status as a critical confounder

A pivotal finding of this systematic synthesis is the identification of Hormone Receptor (HR) status as the primary confounding determinant of survival outcomes within the HER2-low population. Our analysis highlighted that the clinical behavior of HER2-low breast cancer is not monolithic; rather, it is bifurcated by HR expression (4, 13). Specifically, tumors characterized as HR-positive/HER2-low demonstrate a significantly superior prognosis, with a median Overall Survival (OS) extension of 4 to 6 months compared to the HR-negative/HER2-low (triple-negative-like) subgroup (5, 12).

This survival disparity is largely attributable to the inherent biological indolence of luminal subtypes and the availability of diverse endocrine-based therapeutic backbones, such as CDK4/6 inhibitors, which are inaccessible to HR-negative patients (3, 17). In the HR-negative/HER2-low cohort, the absence of both HR signaling and HER2 oncogenic addiction results in a more aggressive clinical phenotype with limited targeted options outside of novel antibody-drug conjugates (ADCs) (2, 15). These findings indicate that HER2-low status should be viewed as a complementary rather than a primary factor in prognostic stratification, where hormone receptor (HR) status remains the dominant driver. Clinical frameworks must, therefore, adjust for HR-mediated confounding to accurately assess the independent contribution of HER2-low expression to long-term survival (12, 18).

### 4.8 Limitations and future perspectives

Despite the comprehensive nature of this systematic review, several limitations must be acknowledged. First, there is significant methodological heterogeneity across the included studies, particularly regarding the assays used for HER2 scoring. The current IHC-based classification was originally designed to identify HER2-positive (amplified) tumors, and its sensitivity in reliably distinguishing HER2-low (IHC 1+ or 2+) from HER2-zero (IHC 0) remains a subject of ongoing pathological debate (1, 2, 28). Furthermore, the inherent subjectivity and variability in HER2 interpretation among pathologists potentially introduce misclassification bias. Such inter-observer discrepancies in identifying IHC 1+ and IHC 2+/ISH-negative status may confound the survival disparities observed across different studies. Relatedly, as discussed in Section 4.4, all included studies reported IHC 1+ and IHC 2+/ISH-negative tumors as a single combined HER2-low group; the lack of IHC-subgroup-stratified survival data across the evidence base is itself a limitation of the current literature that this review could not overcome, and it likely masks clinically relevant prognostic heterogeneity within the HER2-low spectrum (39).

Second, the follow-up duration in several pivotal trials evaluating novel ADCs is relatively immature. While Progression-Free Survival (PFS) data are robust, the long-term Overall Survival (OS) disparities may evolve as more mature data from ongoing phase III trials, such as the DESTINY-Breast series, become available (3, 11). Several of the observational cohorts included in this synthesis also had a median follow-up of under 24 months (**Table 2**), which is insufficient to fully characterize long-term OS in a disease where median survival commonly exceeds 30 months; the immaturity of follow-up data across the evidence base should be considered when interpreting the OS estimates reported here. Furthermore, most included studies are retrospective or *post-hoc* analyses, which inherently carry risks of selection bias and unmeasured confounding, particularly concerning prior lines of systemic therapy. As detailed in Section 2.7.5, the included studies also did not apply a uniform set of covariates (age, tumor stage, treatment line, visceral metastasis) when calculating the reported hazard ratios, which is an additional source of unmeasured confounding that a formal meta-analysis could not have resolved either.

Third, a specific and clinically important form of selection bias relates to differential access to novel ADCs. Because Trastuzumab Deruxtecan and other HER2-targeted ADCs were, for most of the study period covered by this review, available only within clinical trials, in expanded-access programs, or in health systems with reimbursement for these agents, the HER2-low cohorts in several observational studies were effectively selected toward patients who did or did not have access to ADC therapy rather than reflecting the full real-world HER2-low population. This access-related selection bias likely contributes to the discrepancy between the favorable HER2-low outcomes seen in ADC-treated trial populations and the less favorable outcomes seen in real-world cohorts predating widespread ADC availability, and should be distinguished from a true biological survival disadvantage of HER2-low disease.

Fourth, the geographic and ethnic composition of the evidence base is skewed toward North American and European populations; large-scale cohort data from Asian populations, where differences in HER2-low prevalence, molecular subtype distribution, and access to ADC therapy have been reported, remain comparatively scarce among the studies meeting our inclusion criteria. This limits the generalizability of the synthesized survival estimates to Asian and other underrepresented populations, and should be addressed by future large-scale registry studies in these settings.

Fifth, the search strategy, while expanded in this revision to include Embase and the SABCS conference archive (Section 2.3), remains subject to the residual possibility that eligible pharmacology-focused or non-English-language records were not captured; the sensitivity check described in Section 2.3 did not identify additional eligible studies, but cannot fully exclude this possibility.

Finally, HER2 expression is not static: conversion of HER2 status between primary and metastatic or recurrent lesions has been reported in a meaningful proportion of patients, and most of the included studies characterized HER2 status at a single time point (either at diagnosis or at the time of the survival analysis) rather than re-assessing HER2 status longitudinally. Because a proportion of tumors classified as HER2-low at diagnosis may become HER2-zero, HER2-positive, or vice versa over the course of disease, the survival trajectories attributed to a fixed HER2-low classification in this review may not fully capture the dynamic nature of HER2 expression, an issue that is of particular relevance for treatment sequencing decisions involving serial re-biopsy (5, 23).

The clinical management of HER2-low breast cancer is rapidly evolving. Future research must prioritize the development of quantitative HER2 assays, such as proteomics or digital spatial profiling, to more precisely identify patients who will derive maximal clinical benefit from targeted antibody-drug conjugates (14, 15). Additionally, the potential synergy between ADCs and immune checkpoint inhibitors (ICIs) in the HER2-low space warrants further investigation in prospective randomized trials to determine if this combination can further narrow the survival gap between HER2-low and HER2-positive diseases. Finally, understanding the evolution of HER2 expression from primary to metastatic sites will be crucial for optimizing treatment sequencing in an increasingly complex therapeutic scope (5, 18).

## 5 Conclusion

Significant survival disparities exist between HER2-low and HER2-positive breast cancer. HER2-positive disease remains the gold standard for targeted intervention with superior early-line survival. However, HER2-low status is a crucial independent predictor of PFS in the context of ADC therapy. Clinicians should prioritize serial biopsies to accurately capture HER2 status and optimize OS.

## Data Availability

The original contributions presented in the study are included in the article/**Supplementary Material**. Further inquiries can be directed to the corresponding author.

## Supplementary material

Supplementary Table 1 provides the detailed systematic search strategy across PubMed/MEDLINE, Scopus, and the Cochrane Library.

Supplementary Table 2 and Supplementary Figure 1 present the comprehensive methodological quality and risk of bias assessment conducted using the Newcastle-Ottawa Scale (NOS) for observational studies and the Cochrane Risk of Bias (RoB 2.0) tool for randomized controlled trials.

Supplementary Table 3 contains the PRISMA 2020 Checklist, ensuring that this systematic review adheres to the highest reporting standards for clinical oncology research and ensures the transparency of our comparative analysis between HER2-low and HER2-positive survival outcomes.

Supplementary Table 4 summarizes the clinical characteristics and survival endpoints (PFS and OS) of all included studies, providing a granular comparison of outcomes between HER2-low and HER2-positive phenotypes across both landmark clinical trials and real-world cohorts.
