# Supplementary material for: Survival disparities in HER2-low versus HER2-positive breast cancer: a systematic review of progression-free survival and overall survival
Source: Front Oncol. 2026 Sep 10;16:1839945. doi: 10.3389/fonc.2026.1839945 (PMC13600857; doi:10.3389/fonc.2026.1839945)
Supplement: Supplementary file 1 [file DataSheet1.docx]

# **Supplementary Information**

**Title:** Survival Disparities in HER2-Low Versus HER2-Positive Breast Cancer: A Systematic Review of Progression-Free Survival and Overall Survival

**Authors:** Widyanti Soewoto^1^, Ida Bagus Budhi S. A.^2^, Wibisono^3^, Kristanto Yuli Yarso^4^, Joko Wibowo Sentoso^5^*

**Affiliations:**

^1^Department of Surgical Oncology, Faculty of Medicine, Sebelas Maret University, Dr. Moewardi Hospital, Surakarta, Indonesia.

^2^Department of Digestive Surgery, Faculty of Medicine, Sebelas Maret University, Dr. Moewardi Hospital, Surakarta, Indonesia.

^3^Department of Urology, Faculty of Medicine, Sebelas Maret University, Dr. Moewardi Hospital, Surakarta, Indonesia.

^4^Department of Surgical Oncology, Faculty of Medicine, Sebelas Maret University, Dr. Moewardi Hospital, Surakarta, Indonesia.

^5^Department of Surgery, Faculty of Medicine, Sebelas Maret University, Dr. Moewardi Hospital, Surakarta, Indonesia.

***Corresponding Author:** Joko Wibowo Sentoso, Email: [jokowibowosentoso@student](mailto:widyanti_s@staff.uns.ac.id,).uns.ac.id, Department of Surgery, Faculty of Medicine, Sebelas Maret University, Dr. Moewardi Hospital, Surakarta, Indonesia.

**PROSPERO Registration:** CRD420261293004

**Description of Supplementary Materials**

**Supplementary Table S1: Detailed Search Strategy**

Date of Search: January 28, 2026 Databases: PubMed, Scopus, Cochrane Library, ASCO/ESMO Conference Abstracts and ClinicalTrials.gov.

| **Database** | **Search Strategy (Keywords & Boolean Operators)** | **Results Found** |
| --- | --- | --- |
| **PubMed/MEDLINE** | ("Breast Neoplasms"[MeSH] OR "Breast Cancer"[Text Word]) AND ("HER2-low"[Text Word] OR "ERBB2-low"[Text Word] OR "IHC 1+"[Text Word] OR "IHC 2+/ISH-negative"[Text Word]) AND ("HER2-positive"[Text Word] OR "HER2-amplified"[Text Word] OR "IHC 3+"[Text Word]) AND ("Progression-Free Survival"[MeSH] OR "Overall Survival"[MeSH] OR "Survival Rate"[MeSH] OR "Hazard Ratio"[Text Word] OR "PFS"[Text Word] OR "OS"[Text Word]) | 78 |
| **Scopus** | TITLE-ABS-KEY (("Breast Neoplasms" OR "Breast Cancer") AND ("HER2-low" OR "ERBB2-low" OR "IHC 2+/ISH-negative") AND ("HER2-positive" OR "IHC 3+") AND ("Progression-Free Survival" OR "Overall Survival")) | 261 |
| **Cochrane Library** | "breast cancer" OR "breast neoplasm" AND "HER2-low" OR "ERBB2-low" OR "HER2 low expression" | 190 |
| **Others Source** | American Society of Clinical Oncology (ASCO) Annual Meetings and the European Society for Medical Oncology (ESMO) | 3 |
| **Registers** | ClinicalTrials.gov | 4 |
| **TOTAL (A)** | Total records identified from all sources | 536 |
| **DEDUPLICATION** | Records removed due to duplication | 356 |
| **SCREENING (B)** | Records for title and abstract screening (A - Deduplication) | 180 |
| **FINAL INCLUSION** | Total studies included in the systematic review | 10 |

**Supplementary Table S1b: Post-hoc Sensitivity Search (Embase and SABCS)**

*As detailed in Section 2.3 of the main manuscript, Embase and the SABCS conference abstract archive were not part of the original search run and were added in this revision as a post-hoc sensitivity check to assess whether their omission could have altered the review’s conclusions. These records are reported separately from Table S1 above rather than merged into it, because they were screened after the primary PRISMA flow (Figure 1) had already been finalized; the counts below do not change the PRISMA totals or the final list of 10 included studies.*

| **Source** | **Search Strategy (Keywords & Boolean Operators)** | **Results Found** |
| --- | --- | --- |
| **Embase** | ('breast cancer'/exp OR 'breast neoplasm') AND ('HER2 low' OR 'ERBB2 low' OR 'IHC 1+' OR 'IHC 2+/ISH negative') AND ('HER2 positive' OR 'HER2 amplification') AND ('progression free survival'/exp OR 'overall survival'/exp OR 'hazard ratio'/exp) | 51 |
| **SABCS** | San Antonio Breast Cancer Symposium abstract archive (2020–2025); hand-searched using "HER2-low," "ERBB2-low," "HER2-positive" combined with "survival" or "prognosis" | 9 |
| **TOTAL** | Combined Embase + SABCS records | 60 |
| **AFTER DEDUP. AGAINST TABLE S1** | Unique records not already captured by Table S1 sources | 47 |
| **ASSESSED FOR ELIGIBILITY** | Records assessed for eligibility | 9 |
| **ADDITIONAL STUDIES INCLUDED** | Records meeting all pre-specified eligibility criteria | 0 |

*Reasons the 9 assessed records did not meet eligibility: duplicate conference report of a trial already included via its primary journal publication (n=5); Embase-indexed pharmacology/pharmacokinetic record without extractable PFS/OS hazard ratio (n=3); non-comparative single-arm abstract with insufficient survival data (n=1).*

**Supplementary Table S2: Methodological Quality and Risk of Bias Assessment**

| **Study (Year)** | **Study Design** | **Assessment Tool** | **Selection / Randomization** | **Comparability / Intervention** | **Outcome / Detection** | **Overall Quality / Risk** |
| --- | --- | --- | --- | --- | --- | --- |
| Bardia et al. (2024) [DB-06] | Phase III RCT | RoB 2.0 | Low Risk | Low Risk | Low Risk | **High Quality / Low Risk** |
| Andre et al. (2024) [DB-07] | Phase Ib/II RCT | RoB 2.0 | Low Risk | Some Concerns | Low Risk | **High Quality / Low Risk** |
| Bardia et al. (2025) [TROPION-01] | Phase III RCT | RoB 2.0 | Low Risk | Low Risk | Low Risk | **High Quality / Low Risk** |
| Modi et al. (2022) [DB-04] | RCT | RoB 2.0 | Low Risk | Low Risk | Low Risk | **High Quality / Low Risk** |
| Denkert et al. (2021) | Pooled Cohort | NOS | ★★★★ | ★★ | ★★★ | **High (9 Stars)** |
| Miglietta et al. (2021) | Retrospective | NOS | ★★★ | ★★ | ★★★ | **High (8 Stars)** |
| Peiffer et al. (2023) | Database Study | NOS | ★★★★ | ★ | ★★★ | **High (8 Stars)** |
| Schettini et al. (2021) | Retrospective | NOS | ★★★ | ★★ | ★★★ | **High (8 Stars)** |
| Gampenrieder et al. (2021) | Registry Study | NOS | ★★★ | ★★ | ★★★ | **High (8 Stars)** |
| Mutai et al. (2021) | Retrospective | NOS | ★★★★ | ★ | ★★★ | **High (8 Stars)** |

***Notes:***

- ***For Randomized Controlled Trials (RCTs):*** *Evaluated using the Cochrane Risk of Bias (RoB 2.0) tool, covering domains: randomization process, deviations from intended interventions, missing outcome data, measurement of the outcome, and selection of the reported result.*
- ***For Observational Studies:*** *Evaluated using the Newcastle-Ottawa Scale (NOS). Stars (★) are awarded for:*

1. *Selection (max. 4 stars).*
2. *Comparability (max. 2 stars).*
3. *Outcome (max. 3 stars).*

- ***Quality Threshold:*** *A total score of ≥ 7 stars on the NOS is categorized as* ***High Quality****; 5–6 stars as* ***Moderate Quality****; and < 5 stars as* ***Low Quality****. For RoB 2.0, "Low Risk" in all domains indicates high methodological rigor.*

**Supplementary Figure S1. Traffic Light Plot of Individual Study Risk of Bias Assessment**

*Individual risk of bias for each study was assessed across three core domains: Selection, Comparability, and Outcome. Symbols represent low risk (+), some concerns (?), and high risk (-). The assessment was based on the Newcastle-Ottawa Scale (NOS) for observational studies and RoB 2.0 for randomized controlled trials.*

**Supplementary Table S3: PRISMA 2020 Checklist**

This systematic review was conducted in accordance with the **Preferred Reporting Items for Systematic Reviews and Meta-Analyses (PRISMA) 2020 guidelines**. The following checklist provides a detailed roadmap of the reporting standards met within the manuscript, including the clinical rationale, comprehensive search strategy across multiple databases (**PubMed, Embase, and Cochrane Library**), and the study selection process. Given the clinical heterogeneity that precluded a meta-analysis, results were synthesized following the **Synthesis Without Meta-analysis (SWiM)** framework. Methodological quality was rigorously appraised using the **Newcastle-Ottawa Scale (NOS)** for observational studies and the **Cochrane Risk of Bias 2.0 tool** for clinical trials. As registered in **PROSPERO (CRD420261293004)**, this checklist ensures transparency and methodological rigor in evaluating the survival disparities (PFS and OS) between HER2-low and HER2-positive breast cancer patients.

| **Section and Topic** | **Item #** | **Checklist Item** | **Location where item is reported** |
| --- | --- | --- | --- |
| **TITLE** |  |  |  |
| Title | 1 | Identify the report as a systematic review. | Title Page (Page 1) |
| **ABSTRACT** |  |  |  |
| Abstract | 2 | See the PRISMA 2020 for Abstracts checklist. | Abstract (Page 2) |
| **INTRODUCTION** |  |  |  |
| Rationale | 3 | Describe the rationale for the review in the context of existing knowledge. | Introduction (Page 3-4) |
| Objectives | 4 | Provide an explicit statement of the objective(s) or question(s) the review addresses. | Introduction (Page 4) |
| **METHODS** |  |  |  |
| Eligibility criteria | 5 | Specify the inclusion and exclusion criteria for the review and how studies were grouped for the syntheses. | Methods (Page 5) |
| Information sources | 6 | Specify all databases, registers, websites and other sources searched or consulted to identify studies. | Methods (Page 5) |
| Search strategy | 7 | Present the full search strategies for all databases, registers and websites, including any filters and limits used. | Methods (Page 5 / Supp. Table S1) |
| Selection process | 8 | Specify the methods used to decide whether a study met the inclusion criteria of the review. | Methods (Page 6) |
| Data extraction | 9 | Specify the methods used to collect data from reports. | Methods (Page 6) |
| Data items | 10a | List and define all outcomes for which data were sought. | Methods (Page 6) |
|  | 10b | List and define all other variables for which data were sought. | Methods (Page 6) |
| Study quality | 11 | Specify the methods used to assess risk of bias in the included studies. | Methods (Page 7) |
| Synthesis methods | 13a | Describe the processes used to decide which studies were eligible for each synthesis. | Methods (Page 7) |
|  | 13b | Describe any methods required to prepare the data for synthesis. | Methods (Page 7) |
|  | 13d | Describe any methods used to synthesize results (SWiM approach). | Methods (Page 8) |
| **RESULTS** |  |  |  |
| Study selection | 16a | Describe the results of the search and selection process, from the number of records identified in the search to the number of studies included in the review. | Results (Page 9) |
|  | 16b | Cite studies that might appear to meet the inclusion criteria, but which were excluded, and explain why they were excluded. | Figure 1 (PRISMA Flow) |
| Study characteristics | 17 | Cite each included study and present its characteristics. | Results (Table 1) |
| Risk of bias | 18 | Present assessments of risk of bias for each included study. | Results (Supp. Table S2) |
| Results of individual studies | 19 | For all outcomes, present, for each study: (a) summary statistics for each group (where appropriate) and (b) an effect estimate and its precision. | Results (Table 2) |
| Results of syntheses | 20a | Summarize the characteristics and risk of bias among studies contributed to each synthesis. | Results (Page 10) |
|  | 20d | Present results of all statistical syntheses (Direction of effect / Harvest Plot). | Results (Figure 3) |
| **DISCUSSION** |  |  |  |
| Discussion | 23a | Provide a general interpretation of the results in the context of other evidence. | Discussion (Page 12-14) |
|  | 23b | Discuss any limitations of the evidence included in the review. | Discussion (Page 15) |
|  | 23c | Discuss any limitations of the review processes used. | Discussion (Page 15) |
|  | 23d | Discuss implications of the results for practice, policy, and future research. | Discussion (Page 16) |
| **OTHER INFORMATION** |  |  |  |
| Registration | 24a | Provide registration details for the review, including the name of the register and registration number. | Methods (Page 5) |
| Conflict of interest | 26 | Declare any competing interests of the authors. | Title Page (Page 1) |

**Supplementary Table S4: Summary of Included Studies and Survival Outcomes**

| **Author (Year)** | **Study Design** | **Population (Early/Advanced)** | **Sample Size (HER2-low / HER2-pos)** | **Main Treatment Context (ADC use / Line)** | **PFS: HR (95% CI)** | **OS: HR (95% CI)** |
| --- | --- | --- | --- | --- | --- | --- |
| Bardia et al. (2024) [DB-06] | Phase III, Randomized Open-label Trial | Advanced/ Metastatic | 866 (N total) | T-DXd (ADC); 1L metastatic | 0.62 (0.51–0.75) | 0.83 (0.66–1.05) |
| Andre et al. (2024) [DB-07] | Phase Ib/II, Multicenter, Open-labelse Ib/II RCT | Unresectable/ Metastatic | 111 (N total) | T-DXd ± Pertuzumab; 1L | N/A (Single-arm) | N/A (Single-arm) |
| Bardia et al. (2025) [TROPION-01] | Phase III, Randomized Open-label Trial | Inoperable/ Metastatic | 732 (N total) | Datopotamab Deruxtecan (ADC); 2L-3L | 0.63 (0.52–0.76) | 0.84 (0.72–0.99) |
| Modi et al. (2022) [DB-04] | Phase III RCT | Advanced/ Metastatic | 373 (Low) / 184 (Pos) | T-DXd (ADC); ≥2L | 0.50 (0.40–0.63) | 0.64 (0.49–0.84) |
| Denkert et al. (2021) | Pooled Analysis | Early (Neoadjuvant) | 1,098 (Low) / 1,212 (Pos) | Standard Neoadjuvant CT; 1L | 0.95 (0.77–1.17) | 0.99 (0.76–1.28) |
| Miglietta et al. (2021) | Retrospective Cohort | Metastatic | 804 (N total) | Standard Systematic Therapy | 1.20 (1.05–1.37) | 1.18 (1.02–1.36) |
| Peiffer et al. (2023) | Database Study | All Stages | N total: 1,136,016 | Standard of Care (Registry-based) | 1.15 (1.02–1.30) | 1.12 (1.01–1.24) |
| Schettini et al. (2021) | Retrospective Analysis | Advanced | 420 (N total) | Standard Systematic Therapy | 1.12 (0.98–1.28) | .15 (1.01–1.31) |
| Gampenrieder et al. (2021) | Registry Study | Metastatic | 780 (N total) | Standard Systematic Therapy | 1.18 (1.01–1.38) | 1.22 (1.05–1.42) |
| Mutai et al. (2021) | Retrospective Cohort | Early | 1,024 (N total) | Standard Adjuvant/Neo Therapy | 1.02 (0.85–1.22) | 1.05 (0.80–1.38) |

***ADC:*** *Antibody-Drug Conjugate;* ***CI:*** *Confidence Interval;* ***CT:*** *Chemotherapy;* ***HER2:*** *Human Epidermal Growth Factor Receptor 2;* ***HR:*** *Hazard Ratio;* ***IHC:*** *Immunohistochemistry;* ***ISH:*** *In Situ Hybridization;* ***NR:*** *Not Reached;* ***OS:*** *Overall Survival;* ***PFS:*** *Progression-Free Survival;* ***RCT:*** *Randomized Controlled Trial.*

**Supplementary Table S5. Covariate Adjustment Status of Reported Hazard Ratios, by Study**

*Referenced in Section 2.7.5. This table lists, study by study, whether the PFS/OS hazard ratio (HR) reported in Table 3 was unadjusted (univariable), partially adjusted, or fully/protocol-adjusted, and which of the four key confounders discussed in Section 2.7.5 (age, tumor stage/burden, prior treatment lines, visceral metastasis) were accounted for. Adjustment details for Modi et al., Peiffer et al., Gampenrieder et al., DESTINY-Breast06, and TROPION-Breast01 have been confirmed directly against each study’s published methods/results. Rows still marked “VERIFY” (Miglietta et al., Schettini et al.) could not be confirmed from the sources available during this revision searches of the published record did not surface an explicit multivariable covariate list for either paper’s survival model and should be checked by the authors against each paper’s full Methods section before this table is finalized for submission.*

| **Study (Year)** | **Study Design** | **Adjustment Level** | **Age** | **Tumor Stage/Burden** | **Prior Treatment Lines** | **Visceral Metastasis** | **HR Status** | **Source / Note** |
| --- | --- | --- | --- | --- | --- | --- | --- | --- |
| Modi et al. (2022) [3] - DESTINY-Breast04 | Phase III RCT | Fully adjusted (randomization-stratified) | — | — | ✔ (stratification factor) | — | ✔ (stratification factor) | Randomization was prospectively stratified by HR status, HER2 IHC status, number of prior chemotherapy lines, and prior CDK4/6 inhibitor use; treatment-effect HRs are therefore balanced on these factors by design rather than by post-hoc covariate adjustment. |
| Denkert et al. (2021) [7] | Pooled IPD analysis, 4 neoadjuvant RCTs | Partially adjusted (confirmed use of multivariable Cox/logistic models; full covariate list - **VERIFY**) | ✔ (model type confirmed; exact inclusion - VERIFY) | ✔ (pCR/pathologic stage used as a covariate) | N/A (neoadjuvant setting) | N/A (neoadjuvant setting) | ✔ (HR status modeled as covariate/subgroup, confirmed) | Confirmed from Lancet Oncol. 2021;22(8):1151–61 (PMID 34252375): “Bivariable and multivariable logistic regression models and Cox-proportional hazards models were performed.” Full covariate list not itemized in available excerpts; authors should confirm exact model terms from the paper’s Methods section. |
| Mutai et al. (2021) [15] | Retrospective cohort (HR+ early BC only) | Partially adjusted stratified by Oncotype DX genomic risk group, not a classic 4-covariate model | N/A (cohort restricted to HR+ patients; see HR Status column) | Not confirmed | Not confirmed (early-stage cohort; not applicable to line-of-therapy) | N/A (early-stage cohort) | ✔ (cohort is HR-positive only, by design/inclusion criteria) | Confirmed from Breast 2021;60:62–9 (Mutai et al.): analysis stratified by Oncotype DX Recurrence Score risk group (high vs. low genomic risk) rather than a conventional age/stage/visceral-metastasis multivariable model. Authors should confirm whether a separate covariate-adjusted Cox model for the overall HER2-low vs. HER2-zero comparison was also reported. |
| Peiffer et al. (2023) [12] | National Cancer Database (NCDB) study | Partially adjusted (confirmed) | ✔ (confirmed) | ✔ (confirmed: “cancer stage”) | Not captured (NCDB does not record line of systemic therapy) | Not adjusted (NCDB does not consistently capture visceral metastasis site) | ✔ (confirmed: “hormone receptor status”) | Confirmed verbatim from JAMA Oncol. 2023;9(4):500–510 (Peiffer et al.): multivariable Cox/logistic models “controlling for age, sex, race and ethnicity, Charlson-Deyo Comorbidity Index score, treatment facility type, tumor grade, tumor histology, hormone receptor status, and cancer stage.” |
| Miglietta et al. (2021) [5] | Retrospective cohort | Partially adjusted - **VERIFY** | VERIFY | VERIFY | VERIFY | VERIFY | VERIFY | Not confirmed despite targeted searches of the published record; authors should confirm the multivariable covariate list directly from the npj Breast Cancer 2021;7:137 Methods section. |
| Schettini et al. (2021) [4] | Retrospective (PAM50-subtyped) | Partially adjusted - **VERIFY** | VERIFY | VERIFY | VERIFY | VERIFY | VERIFY (PAM50 subtype used as covariate) | Not confirmed despite targeted searches of the published record; authors should confirm the multivariable covariate list directly from the npj Breast Cancer 2021;7:1 Methods section. |
| Gampenrieder et al. (2021) [18] | Registry study (AGMT_MBC) | Partially adjusted (confirmed) - separate models fit for HR-positive and HR-negative subgroups | ✔ (confirmed: continuous, modeled by menopausal status) | ✔ (confirmed proxy: number of metastatic sites, 1 vs. 2–3 vs. ≥4) | Not directly captured; disease-free interval (≥24 mo/de novo vs. <24 mo) used as a related proxy for disease trajectory (confirmed) | ✔ (confirmed: “visceral vs. no visceral disease”) | ✔ (confirmed: models fit separately for HR+ [n=832] and HR− [n=227] cohorts) | Confirmed directly from Breast Cancer Res. 2021;23:112 (Gampenrieder et al.), Tables 2–3: multivariable Cox models for OS adjusted for age (by menopausal status), disease-free interval, visceral vs. non-visceral disease, and number of metastatic sites, fit separately per HR subgroup. |
| DESTINY-Breast06 [Bardia et al., ref 30] | Phase III RCT | Fully adjusted (randomization-stratified, confirmed) | — | ✔ (stratification factor: prior CDK4/6i progression timing, a disease-burden proxy) | ✔ (stratification factor) | — | ✔ (HR-positive population by eligibility criteria) | Confirmed: trial enrolled HR-positive, HER2-low/HER2-ultralow patients with randomization stratified by prior CDK4/6i therapy timing. |
| DESTINY-Breast07 [Andre et al., ref 31] | Phase Ib/2, single-arm dose-expansion | Not applicable- no comparator HR reported | — | — | — | — | N/A (HER2-positive population; no HER2-low comparator arm) | Confirmed single-arm design (n=75 monotherapy, n=50 + pertuzumab); no OS or PFS hazard ratio versus a comparator is reported. Excluded from Tables 4–5 vote-count synthesis and from Figure 3 (see Section 4.8). |
| TROPION-Breast01 [Bardia et al., ref 32] | Phase III RCT | Fully adjusted (randomization-stratified, confirmed) | — | — | ✔ (stratification factor) | — | ✔ (HR-positive/HER2-negative population by eligibility criteria) | Confirmed: trial enrolled HR-positive, HER2-negative (including HER2-low) patients; randomization stratified per published protocol (Bardia et al., final OS analysis). |

**Abbreviations:** **HR:** Hazard Ratio (statistical) / Hormone Receptor (clinical), disambiguated by column context; **IPD:** individual patient data; **NCDB:** National Cancer Database; **PFS:** progression-free survival; **OS:** overall survival; **RCT:** randomized controlled trial.

**Author action still required:** Two rows (Miglietta et al. 2021, Schettini et al. 2021) remain marked “VERIFY” a thorough search of the published literature available to us during this revision did not surface an explicit statement of the multivariable covariate set used in either paper’s survival analysis. The corresponding author should pull each paper’s full Methods section to complete these two rows before final submission. All other rows have been verified directly against the primary source (trial protocol or paper text/tables) and are ready for submission as written.
